# Selective inhibition of tropomyosin-receptor-kinase A (TrkA) reduces pain and joint damage in two rat models of inflammatory arthritis

**DOI:** 10.1186/s13075-016-0996-z

**Published:** 2016-05-04

**Authors:** Sadaf Ashraf, Karyn S. Bouhana, Jed Pheneger, Steven W. Andrews, David A. Walsh

**Affiliations:** Arthritis Research UK Pain Centre, University of Nottingham, Nottingham, UK; Array Biopharma, Boulder, CO USA

**Keywords:** Pain, Inflammation, Nerve growth factor, Tropomyosin-receptor-kinase A, Carrageenan, Knee, Collagen-induced arthritis

## Abstract

**Background:**

Inflammation is an essential component of arthritis pain. Nerve growth factor (NGF) plays a key role in acute and chronic pain states especially those associated with inflammation. NGF acts through tropomyosin-receptor-kinase A (TrkA). NGF blockade has reduced arthritis pain in clinical trials. We explored the mechanisms within the joint which may contribute to the analgesic effects of NGF by selectively inhibiting TrkA in carrageenan-induced or collagen-induced joint pain behaviour. The goal of the current study was to elucidate whether inflammation is central to the efficacy for NGF blockade.

**Methods:**

Rats were injected in their left knees with 2 % carrageenan or saline. Collagen-induced arthritis (CIA) was induced by intradermal injections of a mixture of bovine type II collagen (0.2 mg) and incomplete Freund’s adjuvant (0.2 mg). Oral doses (30 mg/kg) of AR786 or vehicle control were given twice daily after arthritis induction. Ibuprofen-treated (35 mg/kg, orally, once daily) rats with CIA were used as positive analgesic controls. Pain behaviour was measured as hind-limb weight-bearing asymmetry and hind-paw withdrawal thresholds to von Frey hair stimulation (carrageenan synovitis), or withdrawal to joint compression using a Randall Selitto device (CIA). Inflammation was measured as increased knee joint diameter and by histopathological analysis.

**Results:**

Intra-articular injections of carrageenan or induction of CIA was each associated with pain behaviour and synovial inflammation. Systemic administration of the TrkA inhibitor AR786 reduced carrageenan-induced or CIA-induced pain behaviour to control values, and inhibited joint swelling and histological evidence of synovial inflammation and joint damage.

**Conclusions:**

By using two models of varying inflammation we demonstrate for the first time that selective inhibition of TrkA may reduce carrageenan-induced or CIA-induced pain behaviour in rats, in part through potentially inhibiting synovial inflammation, although direct effects on sensory nerves are also likely. Our observations suggest that inflammatory arthritis causes pain and the presence of inflammation is fundamental to the beneficial effects (reduction in pain and pathology) of NGF blockade. Further research should determine whether TrkA inhibition may ameliorate human inflammatory arthritis.

## Background

Nerve growth factor (NGF) plays a key role in persistent inflammatory pain, is expressed within the inflamed synovium and osteochondral junction, and may contribute to arthritic pain [1–4]. Inflammation, angiogenesis, nerve growth and pain are all interconnected processes [5, 6]. NGF sensitises peripheral nerves and may also stimulate blood vessel and nerve growth into structures such as the articular cartilage, which are not normally innervated [7, 8]. Sensory nerves, in turn, might augment inflammation by releasing neuropeptides [9, 10]. Injection of NGF into rat knees induces pain behavior and synovitis [11]. Inhibiting NGF signalling might therefore have particular benefit in patients with inflammatory arthritis pain, including those with rheumatoid arthritis (RA).

NGF binds to tropomyosin-receptor-kinase A (TrkA) and p75 neurotrophin receptors on sensory nerve terminals. Administration of small doses of NGF can produce pain and hyperalgesia [12, 13], and cause neuronal sprouting and elongation [14]. During inflammation or arthritis, NGF levels rise, and nociceptors consequently become sensitised [15–17]. A recent study shows that patients with advanced osteoarthritis (OA) have increased synovial expression of NGF localised predominantly to fibroblasts and macrophages [18]. Macrophages can express the receptor TrkA [19]. NGF blockade can be achieved using antibodies or TrkA-Ig fusion protein, each of which binds NGF and prevents its interaction with TrkA and p75 receptors. Blocking NGF bioactivity largely prevents effects of inflammation on sensory nerve function [20, 21]. An alternative approach is to inhibit the tyrosine kinase activity of TrkA, thereby preventing signalling after binding of NGF. Pan-Trk inhibition reduces ectopic sprouting of sensory nerve fibres, and bone cancer and skeletal pain in mice [22, 23]. It also significantly reduces thermal hyperalgesia and mechanical allodynia in rats with complete Freund’s adjuvant (CFA)-induced paw inflammation [24].

NGF is an attractive target for attenuating chronic arthritis pain [25–28]. Clinical trials of NGF blockade have revealed important benefits in OA [26, 29, 30] but the trials were temporarily halted by the Food and Drug Administration (FDA) following the recognition of rapidly progressive OA in some participants [30]. Although this adverse event appears to be a class effect of NGF blockade, its mechanism remains incompletely understood. Inhibition of signalling through p75 and TrkA receptors may contribute to the effects of NGF blockers, and TrkB and TrkC may contribute to effects of pan-Trk inhibitors. Until recently selective inhibition of TrkA has proved difficult to achieve. AR786 is a novel, orally available selective small molecule inhibitor of TrkA kinase activity, effective at low nanomolar concentrations [31]. We recently demonstrated reductions in pain behaviour in two rat models of OA (meniscal transection and monosodium-iodoacetate-induced), following the administration of the TrkA inhibitor AR786 [31]. To our knowledge no study has specifically looked at whether the inflammatory component in arthritis is essential for the efficacy of NGF blockade via selective TrkA inhibition. Selective TrkA inhibition, therefore, can be an effective analgesic option in inflammatory arthritis.

We hypothesised that the presence of inflammation is central for the beneficial effects of NGF blockade to occur. Selectively blocking NGF activity by targeting its receptor TrkA may reduce pain in part through inhibiting synovial inflammation, although direct effects on sensory nerves are also likely. We used AR786 to explore the contributions of TrkA to pain behaviour and synovial inflammation following intra-articular injection of carrageenan or induction of collagen-induced arthritis (CIA). We observed that in these two well-known models of varying inflammation, selective inhibition of TrkA can reduce carrageenan-induced or CIA-induced pain behaviour by inhibiting synovial inflammation and possibly by direct actions on sensory nerves.

## Methods

### Animals

In vivo studies were performed on either male Sprague Dawley rats (n = 8 per group, 200–220 g; Charles River) in accordance with United Kingdom Home Office regulations and the guidelines of the Committee for Research and Ethical Issues of IASP, or female Lewis rats (n = 10 per group, 125–150 g; Harlan Laboratories) in accordance with the Array BioPharma, Inc. IACUC policies. All animals were housed under standard conditions with food and water *ad libitum* and anaesthetised with isoflurane (2 % in O_2_) prior to injections. All outcome measurements were made by observers blinded to treatment group.

#### Intra-articular carrageenan injection

A single 50-μl intra-articular injection of carrageenan (2 %) dissolved in sterile 0.9 % (normal) saline (pH 7.4) or saline control was given on day 0 into the left knee joints [32–34].

#### Collagen-induced arthritis

Rats were administered three intradermal injections of 0.1 ml of a mixture of 0.2 mg of bovine type II collagen (Elastin Products) mixed equally with incomplete Freund’s adjuvant (Diffco) on days 0 and 6 [35].

### Pharmacological interventions

Rats were dosed with the TrkA selective inhibitor AR786 orally, twice a day at the previously published effective dose of 30 mg/kg in 500 μl or vehicle control (5 % Gelucire) [22, 23, 31]. In the carrageenan model and controls, rats were dosed 1 h prior to and 8 h after the intra-articular injection and then twice daily (each pair of doses separated by 6 h) until the end of the experiment (days 1 or 4). In the CIA experiment, rats were dosed twice daily beginning on day 0 and continuing until day 17. Ibuprofen 35 mg/kg orally, once daily in 0.5 % Tween-80 (critical micellar concentration 1 %), was used as a positive analgesic control.

### Pain behaviour

Pain behaviour was assessed before oral dosing as weight-bearing asymmetry and as punctate allodynia in the hind paw distal to the injected knee, or by paw withdrawal to ankle joint compression. Weight-bearing asymmetry was assessed as the average of five readings from each animal using an incapacitance meter (Linton Instruments, Norfolk, UK), measured as the difference in weight borne between the ipsilateral-treated and contralateral control limb [36]. Punctate allodynia was measured as paw withdrawal thresholds at the ipsilateral and contralateral sides using a series of von Frey monofilaments (Semmes-Weinstein monofilaments (bending forces of 1, 1.4, 2, 4, 6, 8, 10 and 15 g)) by increasing and decreasing the stimulus intensity at each observation time point [37]. Paw withdrawal to ankle joint compression was measured using a Randall Selitto device on day 17 post collagen challenge.

### Inflammation

Joint inflammation was assessed as joint swelling using digital electronic calipers (Mitutoyo, UK), and by histology. Knee swelling was measured at the time of pain behavioural assessments, with values representing differences in knee diameters between the injected and contralateral joints. Ankle diameters were measured at baseline on day 9 after collagen challenge, and then daily until sacrifice at day 17.

Rats were killed by asphyxiation in carbon dioxide, and synovia with patellae from each knee were snap frozen in optimum cutting temperature compound (OCT) over melting isopentane. Hind paws were fixed in 10 % neutral buffered formalin solution for 7 days, then decalcified in 5 % formic acid for 7–10 days and paraffin embedded.

Knee synovial inflammation grade was assessed on haematoxylin-and-eosin-stained sections on a scale of 0 (lining cell layers 1–2 cells thick) to 3 (lining cell layer >9 cells thick and/or severe increase in cellularity) [36, 38]. In the CIA study, paw sections were stained with toluidine blue and scored by a board-certified veterinary pathologist for synovitis, pannus, cartilage damage, bone resorption and periosteal bone formation using a 0–5 scale (0; normal, 5; severe damage) [39–41].

Macrophage infiltration was identified in 5-μm sections of knee synovium by immunoreactivity for CD68 using the mouse monoclonal antibody clone ED1 [42] and the peroxidase-conjugated avidin-biotin-peroxidase complex (ABC) method [43]. Proliferating cell nuclear antigen (PCNA)-immunoreactive CD31-positive cells were taken to identify proliferating endothelial cells (ECs) as a measure of the extent of angiogenesis [44]. Nuclei were counterstained with 4’-6’-diamidino-2-phenylindole hydrochloride (DAPI) [32, 45].

Image analysis was performed by an observer blinded to experimental details using a Zeiss Axioscop-50 microscope (Carl Zeiss Ltd, Welwyn Garden City, UK) and a × 20 objective lens. Transmitted light and fluorescence images of the same field were captured using a 3-CCD camera and analysed using a KS300 image analysis system (Image Associates, Thame, UK) [34]. Macrophage fractional area was defined as the percentage of synovial area that was CD68-positive. EC proliferation index was defined as the percentage of EC nuclei positive for PCNA. For computer-assisted image analyses, four fields per section and one section per case were measured. These numbers were determined in previous experiments [32] to minimise the coefficient of variation, and so that the observed mean lies within ± 12.5 % of the true mean.

### Statistical analysis

Data were analysed using Statistical Package for the Social Sciences v.16 (SPSS inc., Chicago, IL, USA) and graphically presented using Prism v 4 (GraphPad, San Diego CA, USA). Area under the curve (AUC) was expressed in mm/day and calculated for carrageenan-induced synovitis as the integrated product of increase over control knee diameter, and for CIA as the integrated product of the average of the two ankle diameters per rat. Normally distributed data (EC PCNA indices and macrophage fractional areas (logarithmically transformed), incapacitance, paw withdrawal using the Randall Selitto device, and joint diameters) were analysed using one-way analysis of variance (ANOVA). Univariate comparisons were made using Student’s *t* test. Non-normally distributed data were analysed using the Kruksal-Wallis test followed by the Mann-Whitney test to compare two groups. Bonferroni corrections were applied for multiple comparisons. Numerical data are quoted as mean (95 % confidence interval) or median (interquartile range (IQR)) in the text, and, for clarity, graphically as mean ± SEM unless otherwise stated. *P* < 0.05 was taken to indicate statistical significance.

### Reagents

Monoclonal antibody to PCNA (clone PC10) was obtained from DAKO Ltd. (High Wycombe, UK). Biotinylated rat-adsorbed horse anti-mouse antibody and ABC kits were from Vector Laboratories Ltd. (Peterborough, UK). Monoclonal antibodies to rat CD31 (clone TLD-3A12) and to macrophages (CD68, clone ED1) were from Serotec Ltd. (Oxford, UK). Gelucire vehicle was from Gattefosse Corporation (Paramus, NJ, USA). AR786 was provided by Array BioPharma (Boulder, CO, USA). All other chemicals were obtained from Sigma-Aldrich (Poole, UK).

## Results

### Effects of intra-articular injection of 2 % carrageenan on pain behaviour and joint inflammation

**Fig. 1:**
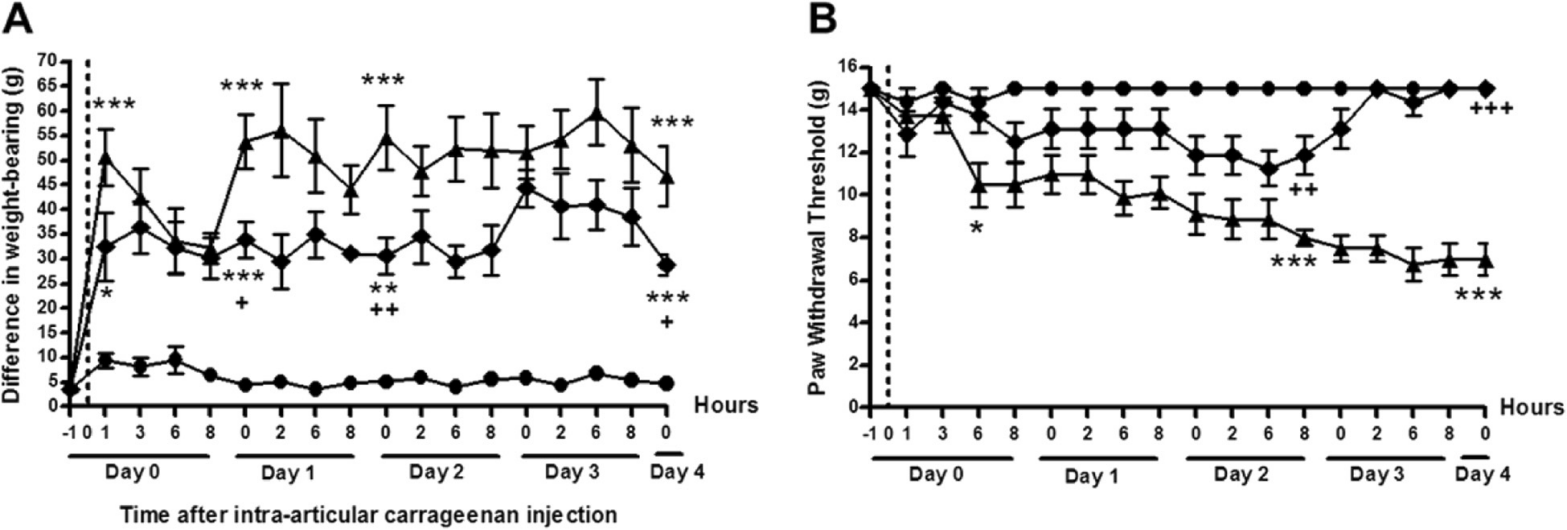
Effects of the selective tropomyosin-receptor-kinase A (TrkA) inhibitor AR786 on carrageenan-induced pain behaviour. Rat knees were injected with either 2 % carrageenan (*triangles* and *diamonds*) or saline (*circles*) on day 0 (*dotted line*). Twice-daily oral dose of 30 mg/kg AR786 (*diamonds*) or 5 % Gelucire vehicle (*triangles*) control was given 1 h prior to and 8 h after the carrageenan injection and then twice daily (each pair of doses separated by 6 h) through day 4. Vehicle-treated carrageenan-injected animals (*triangles*) had increased pain behaviour measured as increased difference in weight-bearing (**a**) and reduced paw withdrawal thresholds (**b**) through 4 days after carrageenan injection compared with saline-injected controls (*circles*). Administration AR786 was associated with reduced pain behaviour, with reduced weight-bearing asymmetry from day 1 and increased paw withdrawal thresholds from day 2. Paw withdrawal thresholds were similar in AR786-treated, carrageenan-injected animals to those in saline-injected (non-synovitic) control levels by day 4. Paw withdrawal threshold was not evoked on the contralateral side. *p < 0.05, **p < 0.01 and ***P < 0.001 compared with saline-injected controls; ^+^ *p* < 0.05, ^++^ *p* < 0.01, ^+++^ *p* < 0.001 compared with vehicle-treated, carrageenan-injected animals

**Fig. 2:**
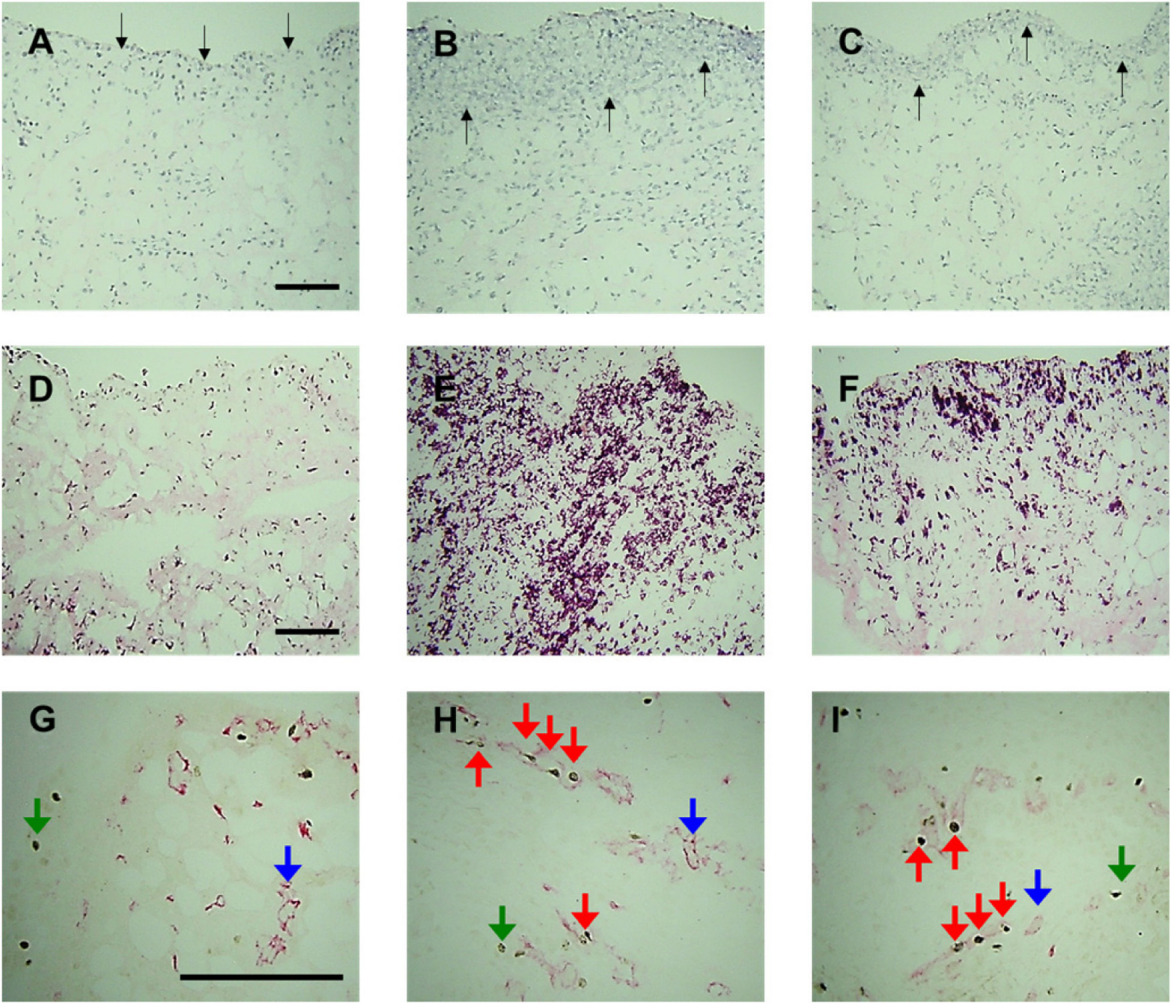
Effects of selective tropomyosin-receptor-kinase A (TrkA) inhibitor AR786 on carrageenan-induced synovial inflammation and angiogenesis. Saline-treated control animals demonstrate normal synovial lining layer thickness/cellularity (**a**), macrophage infiltration (**d**) and endothelial cell (EC) proliferation (**g**). Four days after intra-articular injection of 2 % carrageenan there was an increase in synovial lining layer thickness/cellularity (**b**), macrophage infiltration (**e**) and endothelial cell (EC) proliferation (**h**). Following treatment with AR786 (**c**, **f**, **i**), synovial lining layer thickness/cellularity (**c**) and macrophage infiltration (**f**) were significantly reduced but not to saline control levels (**a**, **d**). EC proliferation was not significantly affected following treatment with AR786 (**i**). Photomicrographs show synovial lining (*black arrows*) and cellularity as indicated by haematoxylin and eosin staining (**a**–**c**), macrophages (*purple/black*) as delineated by immunoreactivity for CD68 (**d**–**f**) and EC (*red*) as delineated by immunoreactivity for CD31 (*blue arrows*), proliferating nuclei (*black*), as delineated by immunoreactivity for proliferating cell nuclear antigen (PCNA) (*green arrows*), and proliferating ECs (*red arrows*), which contain PCNA-immunoreactive nuclei (**g**–**i**). *Bars* = 100 μm

**Fig. 3:**
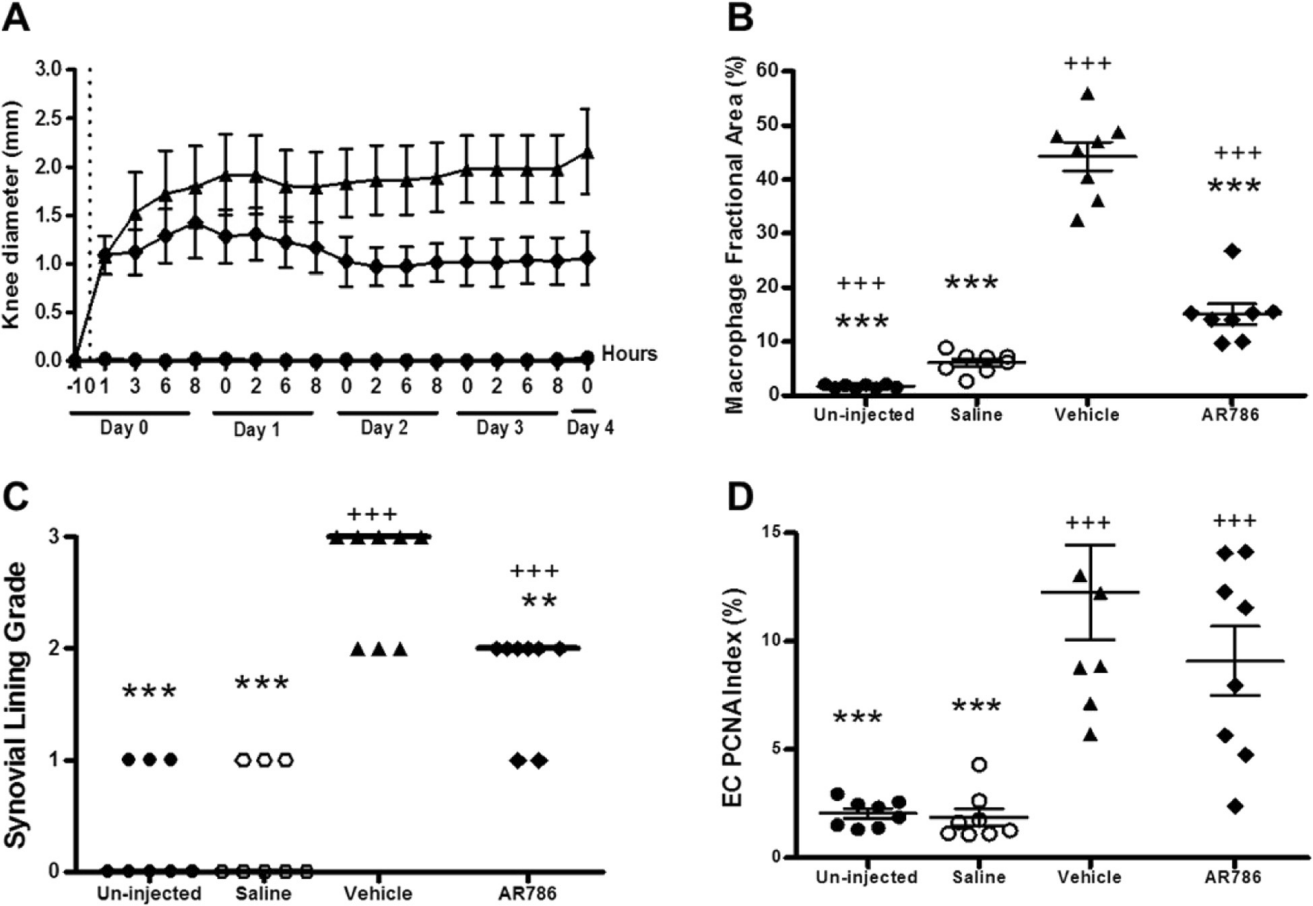
Effects of selective tropomyosin-receptor-kinase A (TrkA) inhibitor AR786 on carrageenan-induced joint inflammation. Rat knees were injected with either 2 % carrageenan (*triangles* and *diamonds*) or saline (*circles*) on day 0 (*dotted line*). Twice-daily oral doses of 30 mg/kg AR786 (*diamonds*) or 5 % Gelucire vehicle (*triangles*) control were given 1 h prior to and 8 h after the carrageenan injection and then twice daily (each pair of doses separated by 6 h) through day 4. Joint swelling (**a**) in carrageenan-injected knees was partially but significantly reduced following treatment with AR786 (*diamonds*) when compared to the vehicle-treated carrageenan-injected animals (*triangles*) (increased AUC over saline-injected, non-inflamed control knees 9.3 (95 % CI 8.4 to 10.1) mm/day versus 15.0 (95 % CI 13.4 to 16.6) mm/day, *p* < 0.001). Four days after carrageenan injection, macrophage infiltration (**b**) and synovial lining layer thickness/cellularity (**c**) were partially reduced, although synovial angiogenesis (endothelial cell (EC) proliferation index) (**d**) was not significantly affected in rats that were treated with AR786 compared with vehicle-treated, carrageenan-injected controls. **p < 0.01, ***p < 0.001 versus vehicle-treated carrageenan-injected animals; ^++^ *p* < 0.01, ^+++^ *p* < 0.001 versus saline-injected (non-synovitic) controls. *Horizontal bars* (**c**) represent median values

Injection of 2 % carrageenan into rat knees induced pain behaviour as measured by hind limb weight-bearing asymmetry (50.6 (37.2–64.1) g) at 1 h compared to saline-injected controls (9.4 (5.6–13.2) g, *p* < 0.001), and weight-bearing asymmetry was maintained through day 4 (Fig. 1a). Carrageenan injection was also followed by a progressive reduction in hind paw withdrawal thresholds to mechanical punctuate stimulation. Significant reductions in hind paw withdrawal thresholds were first seen 6 h after carrageenan injection (10.5 (8.1–13.0) g) compared with saline-injected rats (14.4 (13.0–15.9) g, *p* < 0.05), and paw withdrawal thresholds further decreased through day 4 (Fig. 1b). One hour after carrageenan injection knee diameter was increased (1.1 (0.9–1.3) mm) compared to saline controls (0.02 ([0.0–0.04) mm, *p* < 0.001) and the joints remained swollen through day 4 (Fig. 3a). Synovial macrophage infiltration, synovial lining grade and synovial EC proliferation were each significantly increased 4 days after carrageenan injection (Fig. 2b, e and h and Fig. 3).

### Effects of the selective TrkA inhibitor AR786 on carrageenan-induced pain behaviour and joint inflammation

In order to investigate whether carrageenan-induced pain behaviour and synovitis may be mediated by TrkA receptors, rats were treated with the selective TrkA inhibitor AR786. The inhibitor reduced pain behaviour 24 h after carrageenan injection as measured by hind-limb weight-bearing asymmetry (33.9 (25.1–42.6) g) compared to vehicle-treated, carrageenan-injected animals (53.8 (40.8–66.7) g, *p* < 0.01) (Fig. 1a). The reduction in hind-limb weight-bearing asymmetry was maintained through day 4. Administration of AR786 was also associated with increased paw withdrawal thresholds 2 days after carrageenan injection (11.9 (9.7–14.0) g) compared to vehicle-treated, carrageenan-injected animals (8.0 (7.1–8.9) g, *p* < 0.01), and, by day 4, paw withdrawal thresholds did not differ significantly between AR786-treated, carrageenan-injected animals and saline-injected controls (Fig. 1b).

AR786 partially inhibited carrageenan-induced knee swelling (Fig. 3a). Synovitis measured as macrophage infiltration and synovial lining thickness/cellularity was significantly reduced following treatment with the inhibitor (AR786: macrophage infiltration; 15.1 (10.7–19.5) %, inflammation grade; median score 2 (IQR 1.5–2)) compared to vehicle-treated, carrageenan-injected animals (macrophage infiltration: 44.3 (38.0–50.6) %, *p* < 0.001 and *p* < 0.001, respectively; inflammation grade; median score 3 (IQR 2–3), *p* < 0.05 and *p* < 0.01, respectively) (Figs. 2a–f and 3b–c). No significant reduction in carrageenan-induced synovial angiogenesis was observed following treatment with the TrkA inhibitor (Fig. 2g–i and Fig. 3d).

### Effects of collagen-induced arthritis on pain behaviour and joint pathology

**Fig. 4:**
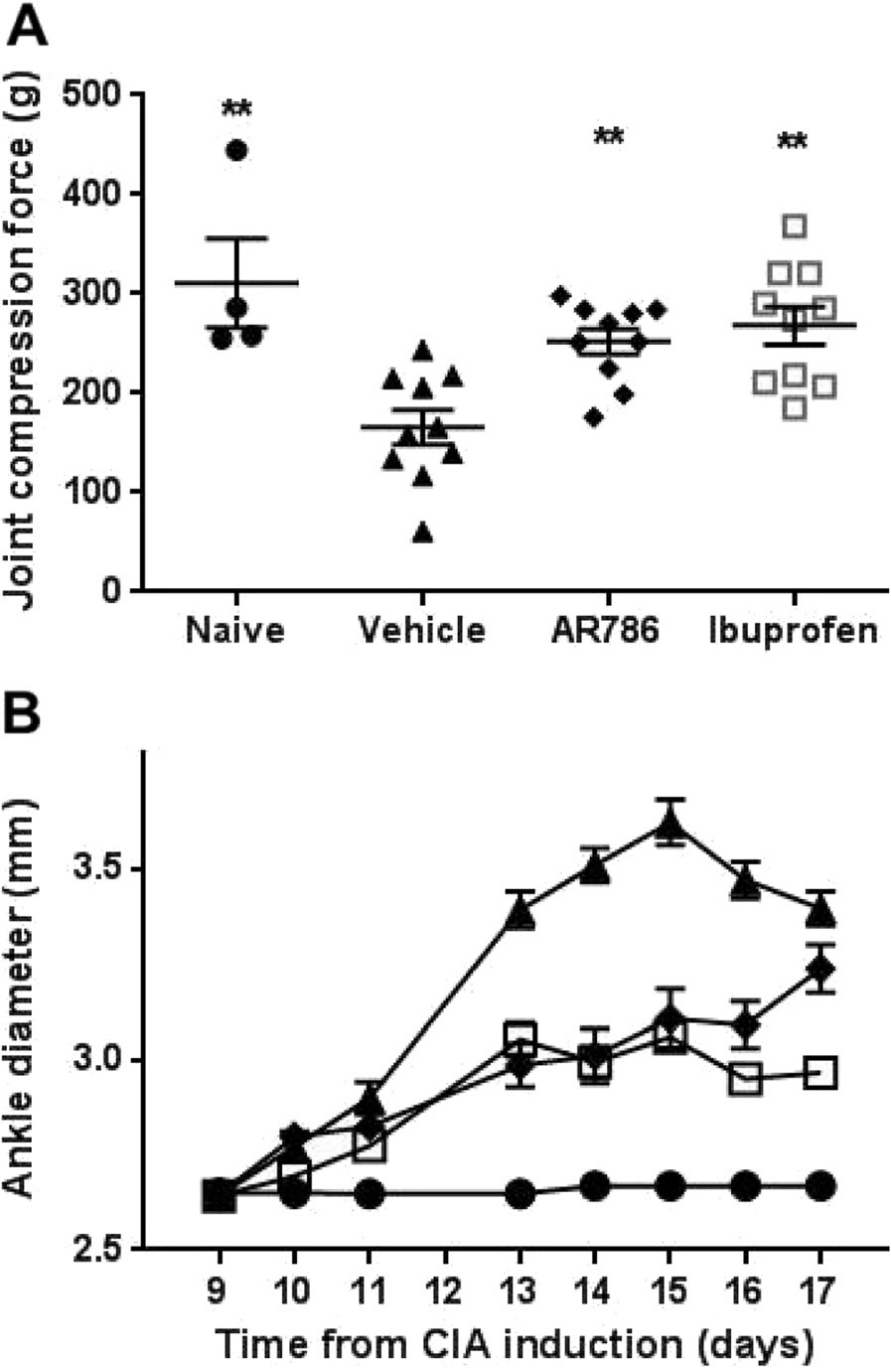
Effects of selective tropomyosin-receptor-kinase A (TrkA) inhibitor AR786 on pain behaviour and ankle swelling following collagen-induced arthritis (*CIA*). Rats were sensitised and challenged to type II bovine collagen in incomplete Freund’s adjuvant. Oral doses of 30 mg/kg AR786, twice daily or 35 mg/kg ibuprofen once daily were administered from the day of collagen challenge. **a** On day 17 pain was measured in the rats using a Randall Selito device. CIA was associated with decreased force required to elicit a withdrawal response. AR786 or ibuprofen each increased the force required to elicit a response to values that did not differ significantly from non-arthritic controls. **b** AR786 (*diamonds*) or ibuprofen (*open squares*) each resulted in significant inhibition of ankle swelling compared to vehicle-treated rats with CIA (*triangles*) (each *p* < 0.005), but the effect did not reach control levels (*circles*) (each *p* < 0.007) (AUC (mm/day) vehicle-treated non-arthritic control 22.6 (95 % CI 21.5 to 23.6), vehicle-treated CIA 33.4 (95 % CI, 31.9 to 34.8), AR786-treated CIA 28.0 (95 % CI 26.1 to 30.0), ibuprofen-treated CIA 27.0 (95 % CI, 26.0 to 28.1); analysis of variance *F* = 32.3, *p* < 0.001. *p < 0.05, **p < 0.01, ***p < 0.001 versus vehicle (5 % Gelucire) controls

**Fig. 5:**
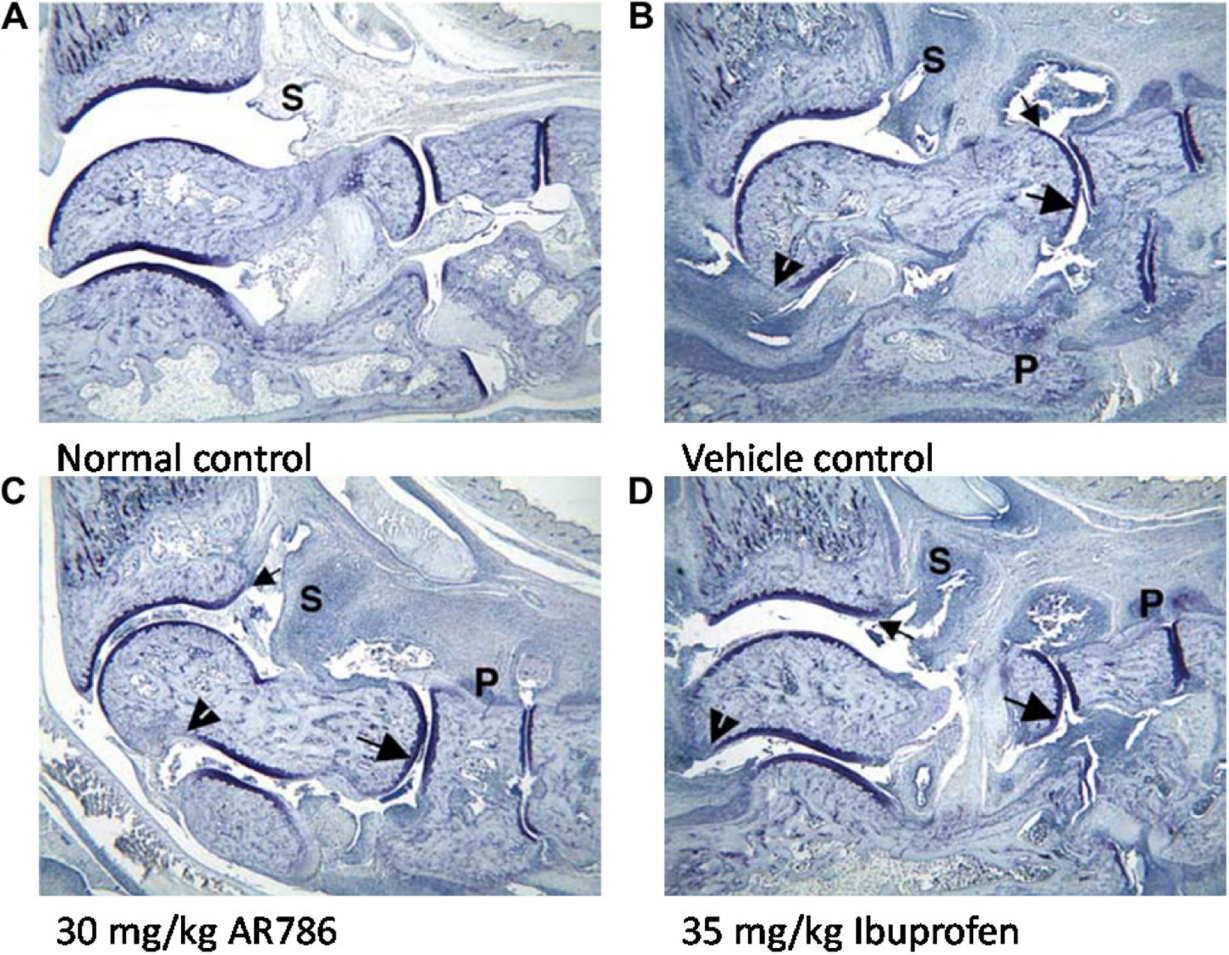
Histological appearances of ankles from rats with collagen-induced arthritis treated with AR786 or ibuprofen. Untreated control animal (**a**) displays normal synovium (*S*), whereas an ankle from a vehicle-treated arthritic animal (**b**) displays severe synovitis (*S*) and moderate cartilage damage (*large arrow*) with mild pannus (*small arrow*) and bone resorption (*arrowhead*). *P* identifies very severe periosteal bone formation. **c** Ankle from an arthritic animal treated with AR786 has marked synovitis (*S*) and mild cartilage damage (*large arrow*) with minimal pannus (*small arrow*) and bone resorption. *P* identifies mild periosteal bone formation. **d** Ankle from an animal treated with 35 mg/kg ibuprofen has severe inflammation (*S*) and moderate cartilage damage (*large arrow*) with mild pannus (*small arrow*) and bone resorption (*arrowhead*). *P* identifies moderate periosteal bone formation. Toluidine blue stain

**Fig. 6:**
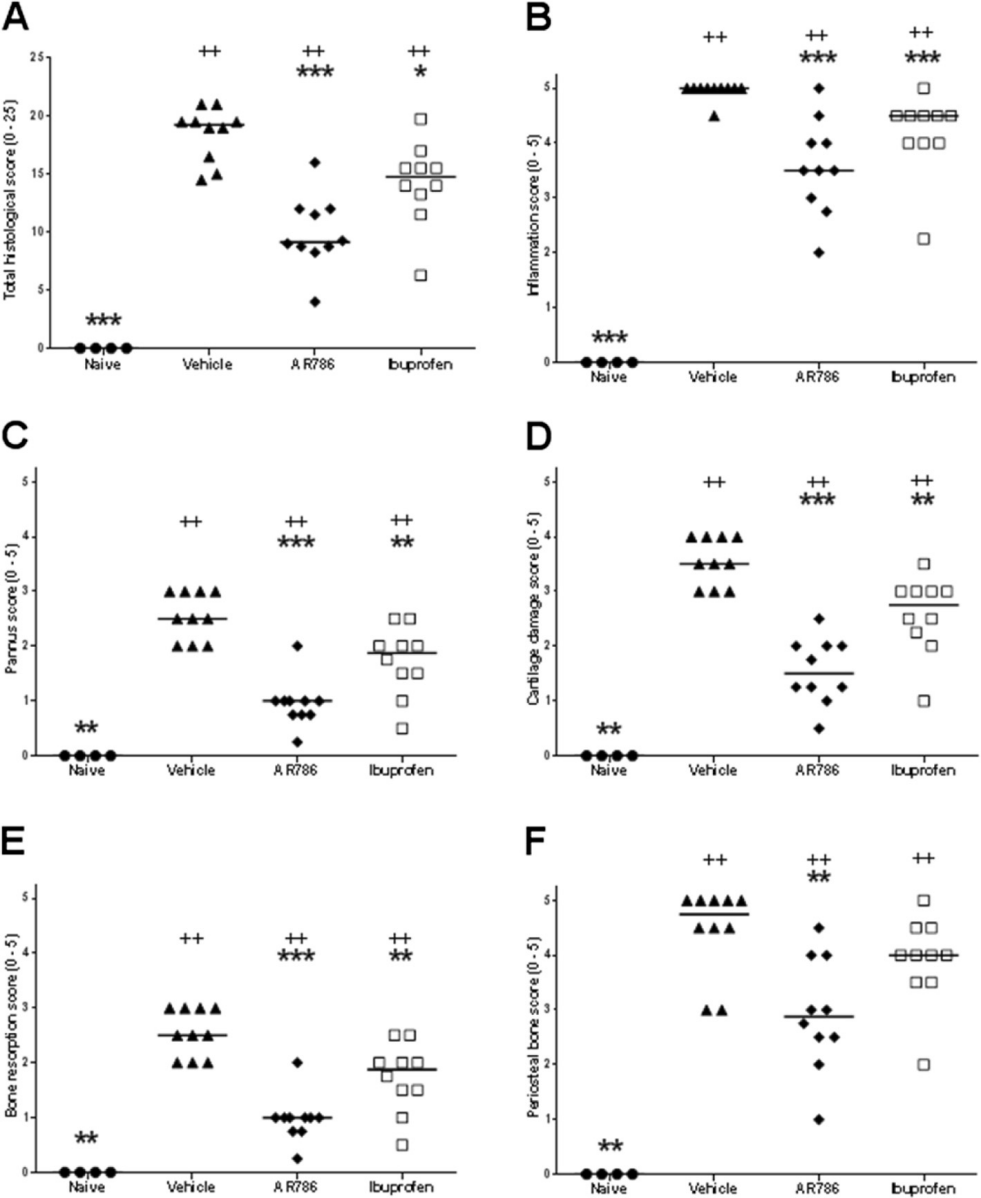
Effects of selective tropomyosin-receptor-kinase A (TrkA) inhibitor AR786 on ankle pathology following collagen-induced arthritis. Collagen-induced arthritis was associated with increased total histological score (**a**), synovial inflammation (**b**), pannus formation (**c**), cartilage damage (**d**), bone resorption (**e**) and periosteal bone formation (**f**). AR786 30 mg/kg orally twice daily from the time of collagen challenge was associated with reduced total histological score and each of its components after 17 days. Ibuprofen 35 mg/kg orally once daily also significantly reduced total histological scores, inflammation, pannus, cartilage damage and bone resorption sub-scores. Kruskal-Wallis statistics all >19, *p* ≤ 0.0002. Comparisons versus vehicle-treated arthritic animals, *p < 0.05, **p < 0.01, ***p < 0.001, and versus naïve controls ^+^ *p* < 0.05, ^++^ *p* < 0.01, ^+++^ *p* < 0.001. *Horizontal bars* represent median values

Paw withdrawal to ankle compression occurred at lower pressures in rats with CIA-induced arthritis than in naïve animals (Fig. 4a). Immunisation with collagen was associated with the expected increases in ankle diameter (Fig. 4b) and histological evidence of synovitis, cartilage damage, bone resorption, pannus and periosteal bone formation (Figs. 5 and 6).

### Effects of the selective TrkA inhibitor AR786 on collagen-induced pain behaviour, inflammation and joint damage

Treatment with AR786 reduced paw withdrawal to ankle compression in rats with CIA-induced arthritis (mean difference −115 (−193 to −36) g, *p* < 0.01 versus vehicle-treated group, Fig. 4a). Reductions in pain behaviour following AR786 were similar to those observed with ibuprofen (mean difference −144 (−223 to −66) g, *p* < 0.001 versus vehicle-treated group).

AR786 inhibited ankle diameter increases from day 13 after collagen challenge (Fig. 4b). Overall, AR786 reduced the increase in ankle diameter observed in vehicle-treated rats with CIA by 47 %, an effect of similar magnitude to that accompanying treatment with ibuprofen. Treatment with AR786 significantly inhibited all histological aspects of disease (Figs. 5 and 6). Treatment with ibuprofen was also associated with reduced histological scores, although scores after ibuprofen remained significantly higher than after AR786 for total score (*p* = 0.03), pannus (*p* = 0.02), cartilage damage (*p* = 0.007) and bone resorption (*p* = 0.02) (Fig. 6).

## Discussion

We have found that the novel TrkA selective inhibitor AR786 reduced pain behaviour and inflammation associated with either carrageenan-induced or collagen-induced synovitis, suggesting it has the effect of reducing both sensitisation and inflammation. This indicates that the beneficial effects of TrkA inhibition may thus be more pronounced during conditions where the presence of inflammation is fundamental to disease mechanism, such as in arthritis [31]. Our findings also provide evidence for the anti-inflammatory potential of TrkA inhibition. TrkA inhibition might therefore offer a novel therapeutic strategy for reducing arthritis pain.

Both intra-articular carrageenan injection and CIA induced pain behaviour in rats. Reduced paw withdrawal thresholds to von Frey hair stimulation indicated allodynia at a site distal to the carrageenan-injected knee. Similarly, reduced pain thresholds in response to mechanical stimulation have been observed distal to, and remote from arthritic joints in human arthritis, reflecting abnormal central processing in nociceptive pathways [46]. Both direct nociception and neuronal sensitisation might contribute to weight-bearing asymmetry in the carrageenan model and to reduced paw withdrawal thresholds to ankle pressure in the CIA model. Reduced pain behaviour following administration of AR786 is consistent with known effects of NGF on neuronal sensitisation [47]. NGF blockade can lead to a reduction in pain behaviour without blocking inflammation [25, 27, 28], as demonstrated using various animal models of inflammatory or non-inflammatory arthritis and different ways of inhibiting NGF. Effects of NGF on receptors other than TrkA (e.g., p75) are incompletely understood, and the different anti-inflammatory effects of TrkA inhibition and NGF blockade might reflect their different modes of action or the specific type of inflammation present in the various animal models. Contribution of the NGF-TrkA pathway to pain behaviour and joint pathology is also evident in models of OA where administration of AR786 reduced the pain and synovitis associated with OA [31]. In this study, administration of AR786 reduced the synovial inflammation grade (measured using haemotoxylin-and-eosin-stained sections) in the monosodium iodoacetate (MIA) model of OA but not in the meniscal transection (MNX) model of OA. These differences may be due to the differing severity or mechanisms of inflammation for the two models, and the more pronounced effects of TrkA inhibition on synovitis in the current study may be largely dependent on the degree or mechanisms of inflammation present. Our current findings extend those previously observed in OA models to indicate that AR786 reduces macrophage infiltration, a specific component of inflammation. Future studies should explore possible additional anti-inflammatory mechanisms in CIA, including any effects on specific immune responses and cytokine release.

Our findings suggest that pain is mediated by TrkA in these acute and persistent inflammation models. Using several different behavioural measures in two different models of varying inflammation we highlight the potential of TrkA inhibition as a novel analgesic strategy in inflammatory arthritis.

Intra-articular carrageenan or CIA injection induced synovitis, which was characterised by joint swelling and increased synovial cellularity, macrophage infiltration and EC proliferation. NGF expression is associated with inflammatory disease activity in RA [48] and NGF/TrkA might contribute to neurogenic inflammation in arthritis [48] by increasing neuronal release of substance P and calcitonin gene-related peptide [7]. Furthermore, NGF might act directly on immune cells, and is a survival and/or activation factor for B cells, eosinophils and synovial fibroblasts [48–51]. Intra-articular injection of NGF increased synovial EC proliferation [11], consistent with pro-angiogenic actions of NGF [4, 49], and with partial inhibition of carrageenan-induced synovial angiogenesis antagonists of neurokinin-1 receptors for substance P [52]. However, NGF alone is a weak inflammogen [50] and TrkA inhibition only partially inhibited synovitis in the current study, indicating that other factors are also important, as previously demonstrated for TNFα and IL-1 [39, 40].

AR786 had disease-modifying effects in the rat CIA model. These data were not entirely expected because an anti-NGF antibody was previously shown not to reduce joint damage in the Freund’s-adjuvant-induced model of inflammatory arthritis (AIA) [28]. Administration of AR786 also does not significantly affect osteochondral pathology in models of OA [31]. Differences between studies may reflect different mechanisms of inflammation in OA, AIA [39, 51, 53] and CIA [39, 54], although all of these models display pain behavior, which is sensitive to inhibition of the NGF/TrkA pathway. The benefits of AR786 for histopathological endpoints in the CIA model are similar to those seen with TNF-alpha blockade, IL-1RA or methotrexate [39, 40], although further research would be required to determine the potential of TrkA inhibition for RA disease modification.

Our interpretations are subject to several limitations. AR786 was shown through extensive testing to be highly selective for TrkA [31], but we cannot completely exclude effects on other molecular pathways. Further research would be required to explore the possible effects of AR786 in established CIA or other models of human inflammatory arthritis, and the precise cellular mechanisms by which AR786 might inhibit inflammation. However, similarities between reductions in pain behaviour in the current study, and findings with agents that block NGF, both in animal models and in man, support the selection of the NGF/TrkA pathway as a target for arthritis pain. Animal models only approximately reflect mechanisms of human arthritis, and the precise mechanisms of arthritis pain remain uncertain, both in our rat models, and in RA [52]. However, our findings support further development of TrkA inhibitors for the treatment of inflammatory arthritis pain. NGF blockade has been associated with adverse events in clinical trials, notably, but rarely, accelerated osteoarthritic structural damage [55]. The underlying mechanisms are currently unknown and although histological analyses in the current study suggested beneficial rather than harmful effects on joint structure, more detailed toxicology studies are warranted. It remains to be determined whether inhibition of TrkA will fulfil the promise of early clinical trials of NGF blockade, whilst avoiding adverse events.

## Conclusions

In two separate models of varying knee pain, joint pathology and inflammation, we have demonstrated that NGF receptor TrkA inhibition using AR786 can reduce pain behaviour, joint damage and synovial inflammation. Our data suggest for the first time that TrkA inhibitors exert enhanced therapeutic benefit if inflammation is one of the core mechanisms by which the disease progresses. TrkA inhibition in our study, therefore, showed therapeutic potential in models of painful knee inflammation.

## Abbreviations

AIA: adjuvant-induced arthritis
CFA: complete Freund’s adjuvant
CIA: collagen-induced arthritis
EC: endothelial cell
IL: interleukin
NGF: nerve growth factor
OA: osteoarthritis
PCNA: proliferating cell nuclear antigen
RA: rheumatoid arthritis
TNF: tumour necrosis factor
TrkA: tropomyosin-receptor-kinase A
